# Evolution of Microplastics Released from Tea Bags into Water

**DOI:** 10.3390/polym17192700

**Published:** 2025-10-07

**Authors:** Alexander A. Yaroslavov, Anna A. Efimova, Tatyana E. Grokhovskaya, Anastasiia G. Badikova, Vasily V. Spiridonov, Denis V. Pozdyshev, Sergey V. Lyulin, Jose M. Kenny

**Affiliations:** 1 Department of Chemistry, M.V. Lomonosov Moscow State University, Leninskie Gory 1-3, 119991 Moscow, Russia; yaroslav@belozersky.msu.ru (A.A.Y.); ephimova@genebee.msu.su (A.A.E.); groch@genebee.msu.ru (T.E.G.); badikova10_10@mail.ru (A.G.B.); vasya_spiridonov@mail.ru (V.V.S.); 2 Yaroslav-the-Wise Novgorod State University, B. St. Petersburgskaya Str. 41, 173003 Veliky Novgorod, Russia; 3 Belozersky Research Institute of Physico-Chemical Biology, M.V. Lomonosov Moscow State University, Leninskye Gory 1-40, 119992 Moscow, Russia; denispoz@belozersky.msu.ru; 4 European Center for Nanostructured Polymers (ECNP), Loc. PentimaBassa, 21, 05100 Terni, Italy

**Keywords:** microplastics, nanoplastics, polymer degradation, tea bags, dynamic light scattering, laser microelectrophoresis, surface charge, cytotoxicity

## Abstract

Eight different types of tea bags were investigated in this work using dynamic light scattering, electrophoretic mobility and nanoparticle tracking analysis methods to determine the concentration and size of released particles from the bag materials at different temperatures and times. Infrared spectroscopy and calorimetric methods confirmed that the bag material consisted of synthetic (nylon or polypropylene) or natural polymers (cellulose). The size of the released particles lies in the range of 200 nm^–1^ µm with an initial bimodal distribution and with an average diameter of about 600 nm. The concentration of released particles increases with increasing temperature and brewing time. The released particles of synthetic polymers remain quite stable and are not affected by natural enzymes, while cellulose particles are easily degraded by the proteolytic complex Morikrase. When analyzing the electrophoretic mobility, it was found that the released particles have a negative surface charge, which probably determines the absence of cytotoxicity established on the epithelial cell line Caco-2 even at the maximum values of the observed particle concentrations (14 × 10^9^ particle/L for synthetic polymers and 170 × 10^9^ particle/L for cellulose).

## 1. Introduction

The problem of microplastic pollution is increasingly attracting public attention, at least since 2004 [1], and went global in March 2022 when the General Assembly of the United Nations Environmental Program (UNEP) passed a resolution calling for an international legally binding instrument to prevent plastic pollution [2]. The issue has a particular focus on the degradation of used polymers, the formation of small polymer particles from them and their impact on human health. According to the accepted definition, synthetic polymer particles smaller than 5 mm, including particles in the nanometer size range, are called microplastics (MPs) [3,4,5]. Additionally, much of the health impact of microplastics can be attributed to the transport of toxic pollutants by polymer particles [6,7,8].

Polymers are generally chemically inert compounds with widely appreciated high-performance properties. However, the amount of microplastics in nature continues to increase, mainly because most of the produced polymers are very cheap, have a short useful life and are discarded and distributed uncontrollably into the environment after use, accompanied by a continuous increase in primary polymer production [9]. Given the rather slow degradation of most polymer products in normal environmental conditions, the formation of small particles with hazardous concentrations from used polymers must be considered a delayed problem that could become significantly more serious in the near future if there are no decisions on control strategies including, among others, mandatory collection and recycling of the polymer wastes already distributed in the environment.

Despite the fact that MP danger for human health is rather potential and mostly proven in laboratory conditions, where model “engineering” particles are usually considered to have an overrated concentration in comparison to the concentration that humans face in reality, such research approaches and related results are extremely important [10,11,12].

MP’s danger to human health is based mainly on the similarity in size to other small particles with proven toxicity [5,13] and on the huge and growing number of studies of various model living systems, conducted primarily in laboratory conditions [11]. State-of-the-art problems include (1) the correct estimation of real human consumption of MPs, mostly accidentally, via inhalation of air or consumption of MP-containing beverages and food products [5,14,15], characterized by a wide range of data that vary from source to source and change over time [16,17], and (2) the increasing use of new experimental methods to identify and characterize MPs in a given system [18].

A separate issue is the release of MP particles when heating plastics in contact with food. When making a final decision on the toxicity of the MP particles themselves, depending on their chemical structure, size and concentration, the question may also arise about the need to review the permitted types of polymers for food packaging or the limits of the temperature range for their use. Recently, data on MP particles release when heated have been obtained for polypropylene bottles used to prepare food formulations for feeding infants [17], hot drink cups made of LDPE [19] and tea bags made of nylon (polyamide) or polyethylene terephthalate [20]. In an interesting and relevant study on the release of microplastics from brewed tea bags [20], the presence of MP particles was analyzed using scanning electron microscopy (SEM), and their composition was confirmed by X-ray photoelectron spectroscopy (XPS) and Fourier transform infrared spectroscopy (FTIR) methods. The number of particles released from a tea bag in 5 min at 95 °C was 11.6 billion microplastics and 3.1 billion nanoplastics.

The results of this research, on the one hand, provide additional examples of polymer products capable of producing microplastic directly, bypassing the intermediate stages of gradual grinding of the product. On the other hand, these results raise new questions about further evolution of microplastics from the tea bags. What happens after the bags have been in the water for more than 5 min? Does water temperature affect the release of particles from tea bags? Tea bags are known to be made from cellulose or from synthetic polymers. Is there any difference in the behavior of both? Are micro- and nano-sized particles resistant to aggregation? If so, what factors ensure their sustainability? Answering these questions will provide a better understanding of how to properly handle tea bags, especially if MP particles are found to have significant health effects in the future.

The most commonly used analytical methods for detecting MP particles in various systems include Fouriertransform infrared spectroscopy (FTIR) and Raman spectroscopy, gas chromatography with pyrolysis (a destructive method of investigation), optical microscopy and scanning electron microscopy (SEM) [14,18,21,22]. However, there are other relatively simple but informative methods that are not as widely used: dynamic light scattering and laser microelectrophoresis. The first allows controlling the size of MP particles in the critical range, several microns and lower, which, according to numerous publications [23,24,25,26,27,28], poses the greatest danger to living organisms. The second gives information about the electrophoretic mobility (EPM) of micro-sized particles, associated with their surface charge, which largely determines the aggregative stability of particles in aqueous environment and their ability to electrostatically bind various compounds [29]. The latter determines the interaction of MPs with toxic compounds affecting the activity of toxins and the mechanisms of their biological action. The surface charge also determines the interaction of MPs with cells.

To answer the questions raised above, the present study considers a set of different tea bags made of synthetic polymers and natural cellulose. Tea bag parts were soaked in water at different temperatures, after which the release of micro- and nanoscale particles into the aqueous solution and the evolution of their concentration and dimensions as a function of time were recorded. Moreover, the particle EPM and surface charge density were measured. Obtained results were complemented by the toxicity testing of tea bag-released MPsinthe epithelial intestinal model cell line Caco-2. This research addresses for the first time the dynamics of particles released from the tea bags, showing that the final state of the system largely depends on the material of the tea bags and estimates the biological effect of the released particles. The ease of particle preparation, their small size and high surface charge make the tea bag-produced particles a promising model for investigating the behavior of MP particles including those loaded with toxic substances. Such systems represent a more realistic analogue of MPs in the environment.

## 2. Materials and Methods

### 2.1. Materials

Eight tea bag samples (with assigned numbers from I to VIII, under which they are described below) from different manufacturers were considered in the study.

To determine the EPM of polymer particles the water suspensions were titrated with a water suspension of a cationic polymer poly(N-ethyl-4-vinylpyridinium) bromide (PVP) (see details in Procedure S1).

Biodegradation of released tea bag particles was initiated by the addition of the proteolytic complex Morikrase («Trinita LTD», Moscow, Russia), which is a mixture of enzymes capable of cleaving ester, peptide and amide bonds. Morikrase is active within a pH range of 5.5–9, with an optimal pH of 7.5 [30].

Sodium chloride, sodium hydrophosphatedodecahydrate Na_2_HPO_4_*12H_2_O and sodium dihydrogen phosphate NaH_2_PO_4_*2H_2_O (Chimmed, Moscow, Russia) (all chemically pure grade) were used as received, and a 10^–2^ M buffer solution was prepared by weighing.

To prepare solutions, double-distilled water was used, which was additionally treated by passing through a Milli-Q system (Millipore, Burlington, MA, USA)) equipped with ion-exchange and adsorption columns and a 0.22 μmpolyethersulfone membrane filter to remove organic/inorganic particles and bacteria.

### 2.2. Methods

Mean hydrodynamic diameter (D_h_) of polymer particles was measured by dynamic light scattering (DLS) at a fixed scattering angle (90°) in a thermostatic cell with a Brookhaven Zeta Plus instrument (Brookhaven Instruments, Nashua, NH, USA. Software (DynaLS software package (version2.5)) provided by the manufacturer was employed to calculate diameter values (error ±7%). Electrophoretic mobility (EPM) of polymer particles was determined in a thermostatic cell by laser microelectrophoresis by using a Brookhaven Zeta Plus instrument with the corresponding software (error ±5%).

Solution pH was measured with a Radiometer pHM 83 pH meter (Copenhagen, Denmark) equipped with a P1041 glass electrode and a K4041 calomel reference electrode (error ±0.02 units).

The solution conductivity was determined with a Radiometer CDM 83 conductometer (Copenhagen, Denmark) equipped with a PP1042 platinum electrode (error ± 0.01 units).

Identification of polymers in the tested tea bags was performed by IR spectroscopy with an IR-spectrometer Specord M-80 (Carl Zeiss, Oberkochen, Germany) as well as differential scanning calorimetry (DSC) and thermogravimetry (TG) using a Mettler Toledo thermal analyzer TA400 (Greifensee, Switzerland) (see details in Procedure S2).

Nanoparticle tracking analysis (NTA) was conducted using a ZetaView PMX420-QUATT instrument (Particle Metrix GmbH, Inning am Ammersee, Germany), while the data were analyzed by software ZetaView NTA.

All experiments were carried out in quadruplicates.

### 2.3. Tea Bag Preparation for the Analysis

The tea bags were cut with metal scissors to remove the tea leaves. The empty tea bags were thoroughly washed by deionized water and dried at room temperature. The resulting material was cut into 3 cm × 3 cm squares, which were used in the experiments described below. The term “tea bag” in the text below refers to a square of the specified size.

### 2.4. Evaluation of Cytotoxicity

The cytotoxicity of the polymer particles towards Caco-2 cells was evaluated using a methyl tetrazolium blue assay. The tetrazolium dye MTT, which enters living cells and is attacked by redox enzymes, is reduced to formazan and precipitates as dark blue crystals, whereas in dead cells this transformation does not occur (see details in Procedure S3). All experiments were carried out in quadruplicates.

## 3. Results and Discussion

Firstly, the identification of polymers in the tested tea bags was carried out using three independent methods: IR spectroscopy, differential scanning calorimetry (DSC) and thermogravimetry (TG). The first shows functional groups and other fragments in polymers, thereby establishing their composition. The second allows forcontrolling the change in the heat capacity of the material depending on temperature, in particular, for crystallizable polymers, and it is possible to determine the melting temperature (T_melt_) [31,32]. The third can help to investigate physical and chemical transformations accompanied by a temperature-induced mass change (decomposition) of the polymer (T_decomp_), which is specific for a particular polymer.

The IR spectra for all eighttea bags used in the study and their interpretation (see details in Figure S1), which allowed us to identify Sample I and Sample II as polyamide, Sample III and Sample IV as polypropylene and Samples V-VIII as cellulose.The experimental DSC and TG curves for several tea bags (Samples I, III, VI and VII) differed in their chemical composition (see details in Figure S2) and were compared with tabular data in order to identify the polymers in the analyzed samples. The entire list of tested tea bags consisting of eightsamples is presented in Table 1 with the corresponding T_melt_ and T_decomp_ values that confirm the results of the IR tests.

All samples contained additives whose composition was not identified; their content ranged from 5 to 15 wt.%; this assessment was made using the weight fraction of impurities represented by the area of small peaks in the TG curves. Synthetic tea bags from polyamide and polypropylene contained fewer additives than natural cellulose tea bags. Visually, the tea bags were distinguishable: pyramids from synthetic polymers and flat bags from cellulose.

Squares of tea bags by size 3 cm × 3 cm were placed in glasses; distilled water was added to different samples at three different temperatures (20; 50 and 100 °C) and the average particle size in the solutions was determined at different times using the dynamic light scattering (DLS) method. This relatively simple and inexpensive method measures the Brownian motion of macromolecules in solution and relates this motion to the hydrodynamic diameter D_h_ (size) of the particles. DLS allows direct control of particle size in solution without preliminary sample preparation as in various microscopy options.

As the first step, three types of tea bags, from polyamide (Sample I), polypropylene (Sample III) and cellulose (Sample VI), were placed in water for 5 min, as described in the pioneering paper by Hernandez et al. [20], while the soaking was performed at 20, 50 and 100 °C. After removal of the tea bags, an average size of particles in the solution was monitored with DLS (see details in Table S1), which showed reliable results only for polyamide particles at 100 °C and cellulose particles at 50 and 100 °C. In most cases, the particle concentration was too low to be detected by DLS. Therefore, the time of tea bag soaking was extended up to 1 h. The experiments were carried out in two modes. According to the first experiment, hereafter referred to as “short-term” tea bags soaking, tea bags were left in water for 1 h, then removed and not used further. The average particle size in the solution after bag removal was monitored over several days, while the temperature in the glass was kept constant at the three temperatures tested (20 C, 50 C and 100 °C). In the second experiment, the tea bags were kept in water at a given temperature for several days and the solution was periodically tested to measure particle size. This variant was defined as “long-term” tea bags soaking. The scheme of “short-term”(a) and “long-term” tea bags soaking(b) experiments is shown in Figure S3.

The concentration of particles in solution was determined using nanoparticle tracking analysis (NTA). This method allows visualizing the Brownian motion of micro-sized particles [39]. Two types of tea bags were tested from polyamide and cellulose. The experiments were carried out in the “short-term” soaking mode, when 50 °C water was added to the tea bag. After 1 h of soaking corresponded to the “short-term” tea bags soaking procedure, the tea bag was removed and the particle concentration was determined. The solution without tea bags was left for 7 days at 50 °C and the particle concentration was measured again. The concentration of polymer particles in both experiments was in the order of 10–100 billion pieces per liter (see details in Table S2). For synthetic polyamide this value slightly increased with time, while for the cellulose sample it decreased to “zero”. It should be noted that the results on polymer tea bags are of the same order to those already reported in the scientific literature [20].

**Figure 1:**
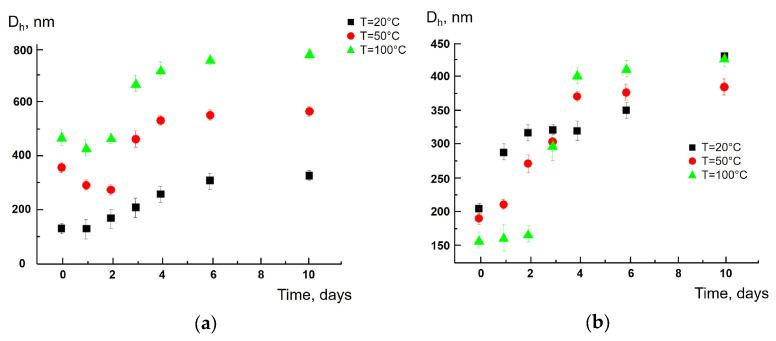
Average size of polyamide particles (Sample II) vs. soaking time at 20, 50 and 100 °C during “short-term” tea bags soaking (**a**) and “long-term” tea bags soaking (**b**).

Below are several examples that illustrate the size of polymer particles of different chemical natures obtained in an aqueous solution. The size, mean hydrodynamic diameter (D_h_), was measured by dynamic light scattering. In the control experiment, distilled water did not show the autocorrelation function thus indicating no scattering particles in the sample. This proved that the results described below were indeed due to particles released to the water from the tea bags. Figure 1a,b reflect “D_h_ vs. time” plots for the particles from the nylon pyramids via the “short-term” and “long-term” tea bag soakings. In both cases, a gradual increase in particle size was found over time: 10 days after the size of particles was 1.5–2 times larger than the size of particles at the beginning of the experiments. When the “short-term” tea bag soaking was carried out (a), higher temperatures resulted in larger particle sizes. At the “long-term” tea bag soaking (b), three “D_h_ vs. time” curves overlapped each other. The “D_h_ vs. time” dependencies were also obtained for synthetic tea bags composed of polypropylene (see details in Figure S4). In this case, both temperature and time had only a minor effect on the size of polymer particles.

**Figure 2:**
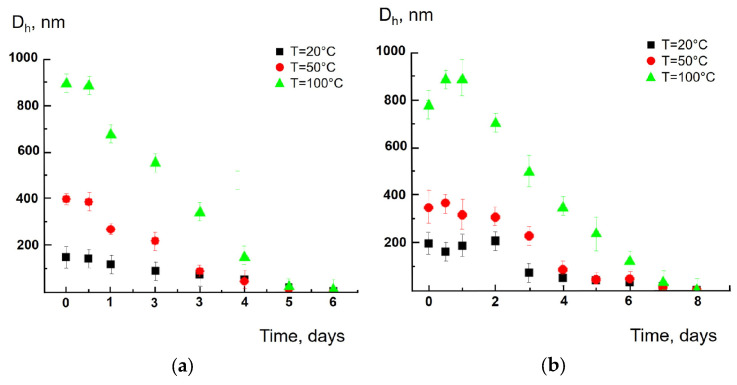
Average size of cellulose particles (Sample V) vs. soaking time at 20, 50 and 100 °C during “short-term” tea bag soaking (**a**) and “long-term” tea bag soaking (**b**).

Particles produced from cellulose tea bags demonstrate quite different behaviors. At the “zero time” moment, the average size of polymeric particles was in the range from 200 to 1000 nm, with the higher the temperature, the larger the particle size (Figure 2a,b). The size gradually decreased with time, and the particles became undetectable 7–9 days after the experiment. In another example, a faster decrease in particle size with time and a slight effect of temperature on the size change kinetics is observed (see details in Figure S5).

Thus, the average size of synthetic and cellulose particles changed with soaking time in opposite directions: in the first case their size increased and in the second one the size decreased to the level of a few nanometers. Additional experiments carried out with synthetic tea bags showed that the released micro-sized particles remained in solution for at least 10 days.

Figure S6 shows TEM images of polyamide particles (Sample II) and cellulose particles (Sample V), 1 and 4 days after “short-term” tea bags soaking; the particles were prepared in 100 °C aqueous solutions. As follows from the photos, the polyamide particles slightly increased their size with time, but the cellulose particles dramatically decreased their size in agreement with the results obtained by the DLS method (see Figure 1a and Figure 2a).

**Figure 3:**
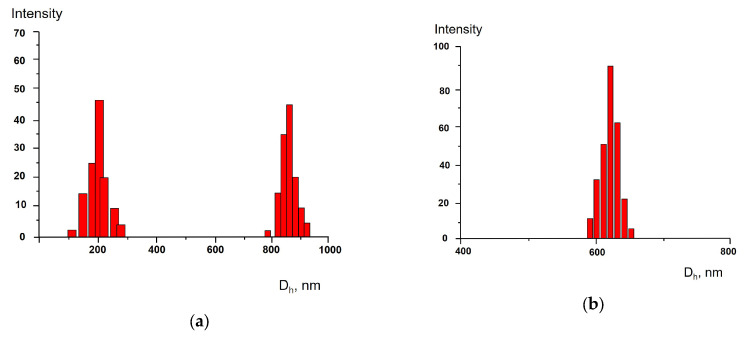
Size distribution of polyamide particles (Sample I) 1 h (**a**) and 7 days (**b**) after soaking at 100 °C; the “short-term” soaking procedure.

More details about the size of the particles were obtained via analysis of size distribution in the tea bag-produced aqueous solutions. As follows from Figure 3a, the size distribution in a solution produced by polyamide-based (synthetic polymer) tea bags showed two modes with maxima at 300 nm (Mode 1) and 900 nm (Mode 2) 1 h after soaking at 100 °C (the “short-term” procedure was applied). Eventually, the contribution of Mode 1 increased with decreasing Mode 2 contribution so that6 days later only a uniform size distribution was detected with a maximum size of approx. 600 nm (Figure 3b). Namely, this value is pointed out in Figure 1 as the average size of the synthetic polymer particles. The bimodal size distribution, obtained by soaking the synthetic tea bags for 5 min and revealed by electron microscopy, has been described earlier [20]. However, the two modes were described as “large particles with size of tens and hundreds of microns” and “small particles with size <1 micron”. The bimodal distribution was also observed, but both modes were in the range of <1 μm. The size distribution within the range <1 micron was not observed and was not discussed in [20]. Our results together with those described in [20] reflect complex processes occurring in tea bags in the surrounding water. The exact mechanism for the transition of the bimodal distribution to the unimodal one is unknown and requires additional research.

The cellulose tea bag, being soaked in water according to the “short-term” procedure, gave the bimodal size distribution as well (see details in Figure S7); both modes decreased in time and 6 days after only one mode remained. At the same time, the average particle size in the system was controlled, which also decreased to an undetectable size, as shown in Figure 2.

The ultimate sizes of the polymer particles, measured 7 days after soaking tea bags with water, are summarized in Table 2. For the synthetic particles, the stationary detectable size is indicated; for the cellulose particles with undetectable sizes, zeros are given.

The “short-term” experiments with the polyamide and cellulose were conducted again, but distilled water was replaced with an extract obtained via traditional tea brewing of the uncut tea bags (see details in Figure S8). The results presented in Figure S8 demonstrate the identity of “D vs. time” plots for particles in distilled water and tea extracts. This allows concluding that there is a little influence of tea leaf components on the release and evolution of microparticles.

At the moment, the mechanism of micro-sized particles release during tea bags soaking in water is still unknown [20,40], as well as it is unknown how microplastics are released from other polymeric materials and products under similar conditions [41]. Polymers are expected to degrade at high temperatures [42] and polyamide nylon is known to be subject to hydrolytic degradation [43]. Hydrolysis could explain the degradation of cellulose tea bags and to some extent nylon tea bags in our experiments, but it is unable to explain the degradation of carbon chain of polypropylene this way. The idea of high-temperature degradation of polymers is also questionable:polypropylene in Sample III degraded with comparable rates at room temperature (20 °C) and at 100 °C. Apparently, particles from the surface of the tea bags samples, “weakly bound” to the polymer matrix, migrate into the solution. The migration is facilitated if there are microdefects on the surface. However, further experiments are needed to elucidate the release mechanism.

It should be emphasized that the soaking of tea bags produces micro- and nano-sized particles, which are a subgroup of microplastics that usually includes particles till 5 mm [20,40].

**Figure 4:**
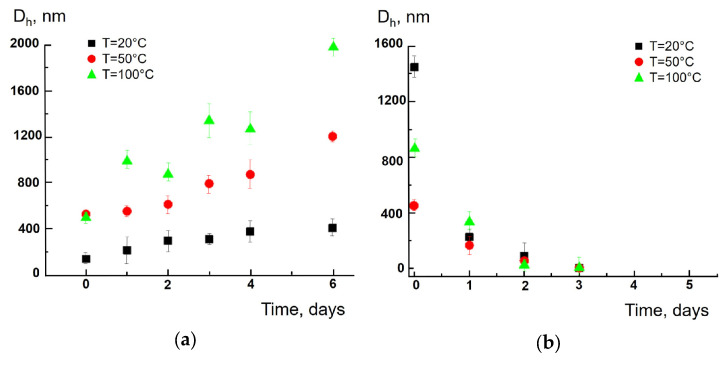
Time-dependent average size of polyamide (Sample I (**a**)) and cellulose (Sample VI (**b**)) particles after the addition of Morikrase at 20, 50 and 100 °C for the “short-term” soaking procedure.

The addition of the proteolytic complex Morikrase, which is a mixture of enzymes that cleave ester, peptide and amide bonds to the tea bag particles produced according to the “short-term” soaking procedure, did not expectedly affect the size of the synthetic polymer particles: the size increased with time as before (Figure 4a), but promoted the degradation of the cellulose particles (Figure 4b).

The measurement of the electrophoretic mobility (EPM) of polymer particles released during the “short-term” soaking, a parameter associated with the surface charge of particles, showed that the particles were negatively charged with EPM from −0.25 to −1.5 (μm/s)/(V/cm) (see details in Figure S9). No correlation between the EMP values and solution temperature was found, and no systematic change in EPM with time was detected.

The negative charge of polymer particles is an important point when discussing their biological effects, since cell membranes also carry a negative charge [44]. Thus, the negative surface charge of polymer particles should prevent their interaction with cells. In turn, this should minimize the possible toxic effect of these particles. Therefore, by controlling the EPM of polymer particles, the number of negative surface groups on a single particle was estimated. For this purpose, a water suspension of polymer particles was titrated with a water suspension of cationic polymer PVP. Binding of PVP on the particle surface was accompanied by neutralization of the particle charge, which altered the EPM of the polymer particle. This approach was previously used to quantify the surface charge of silicate particles [45], polymer microspheres, bilayer lipid vesicles (liposomes) [46] and polymer hydrogels [47]. In particular, it has been shown that the cationic polymer binds quantitatively to anionic multi-charged particles up to complete neutralization of the particle charge [48]. Thus, at the point EPM = 0, the concentration of anionic groups on the particle surface is equal to the concentration of cationic groups in the added polymer. Hence the density of anionic charges on the particle surface can be calculated as

$$\delta =\frac{\left(C_{0}\times N_{a}\right)}{\left(C_{p}\times 4\pi R^{2}\right)}$$

where C_0_ is the molar concentration of the cationic polymer group at EPM = 0, C_p_ is the concentration of a tea bag-produced particles, R is the radius of a single tea bag-produced particle and N_a_ is Avogadro’s number.

Electrophoretic titration of water suspensions of tea bag-produced particles with a water suspension of cationic PVP (see details in Figure S10) allowed us to calculate the anionic group densities of the particles using Equation (1). The results showed extremely high densities of anionic surface groups: 1 Å^−2^ for cellulose and 10 Å^−2^ for s polyamide tea bags. These densities seem too high assuming that the outer boundary of the particles is a relatively smooth surface. A more realistic picture is that the surface of the polymer particles is covered with (nano)cracks that contain anionic groups.

Finally, the cytotoxicity of the tea bag-produced polymer particles was investigated using the intestinal epithelial model cell line Caco-2. This line was chosen because the particles eventually reach the intestine where they can interact with epithelial cells and cause toxic effects. The conventional MTT test was used to detect viable cells. Table 3 shows the viability of Caco-2 cells in the presence of the tea bag-produced polymer particles. The particles demonstrate no cytotoxicity at concentrations of 14 × 10^9^ particle/L for synthetic polymers and 170 × 10^9^ particle/L for cellulose, which were the maximum concentrations reached when the tea bag was “brewed”. This is probably due to the weak interaction of the tea bag particles with Caco-2 cells due to the negative surface charge of the particles, which minimizes the possible toxic effect of the particles [49,50].

## 4. Conclusions

Summarizing, commonly used tea bags consisting of synthetic (nylon or polypropylene) or natural (cellulose) polymers release a huge amount of micron- and nanometer-sized polymer particles during brewing. The results of this work show that the size of the released particles lies in the range of 200nm–1 µm with an initial bimodal distribution and with an average value of about 600 nm. The concentration of released particles increases with increasing temperature and brewing time. However, even the amount of particles released when the tea bags are brewed for an hour does not show signs of cytotoxicity. The detection of these particles was possible using rather simple methods such as DLS, EPM, NTA, DSC, TG and IR spectroscopy. The number of particles detected is on the same order of magnitude as results obtained previously using SEM, XPS and FTIR methods [20], but our work generally detects smaller particles.

Microparticles are released when brewing tea bags made of synthetic polymers remain stable, while particles made of natural polymers easily biodegrade under the action of enzymes (proteolytic complex Morikrase).

The studied particles have a negative surface charge, which makes it possible to assume the absence of cytotoxicity, which was confirmed with epithelial cell line Caco-2. Therefore, the results of this work supports a preliminary conclusion about the safety of using tea bags when brewing tea. However, the reported concentrations of microparticles are high (up to ~170 × 10^9^ particles/L) and further research on the health assessment of daily intake is recommended and planned for future work.

## Figures and Tables

**Table 1:** Identification of polymers in the considered tea bags.

Tea Bag Sample	Experiment		Control		Polymer
	DSC, °T_melt_	TG,°T_decomp_	DSC, °T_melt_	TG, °T_decomp_	
I	220	408	225	400	Polyamide (nylon) [33]
II	210	390	219	394	Polyamide (nylon) [33]
III	168	220	175	290	Polypropylene [34,35]
IV	170	270	175	290	Polypropylene [34,35]
V	-	310	-	350	Cellulose [36,37]
VI	-	343	-	350	Cellulose [36,37]
VII	-	340	-	350	Cellulose [36,37]
VIII	130 admixture	290	-	300	Cellulose [36,38]

**Table 2:** Ultimate (constant) size of particles obtained from tea bags soaked at different water temperatures.

Sample	Ultimate Size, nm		
	°C		
	20	50	100
I	320	580	760
II	330	570	780
III	590	630	610
IV	350	570	680
V	0	0	0
VI	0	0	0
VII	0	0	0
VIII	0	0	0

**Table 3:** Viability of Caco-2 cells in the presence of the tea bag-produced polymer particles.

Sample	Viability of Caco-2 Cells, r.u.
I	0.98 ± 0.03
II	1.04 ± 0.03
III	1.06 ± 0.04
IV	1.02 ± 0.04
V	0.81 ± 0.05
VI	0.98 ± 0.03
VII	1.06 ± 0.01
VIII	0.96 ± 0.01

## Data Availability

The original contributions presented in this study are included in the article/Supplementary Materials. Further inquiries can be directed to the corresponding authors.

## Supplementary Materials

The following supporting information can be downloaded at https://www.mdpi.com/article/10.3390/polym17192700/s1. Procedure S1: PVP synthesis; Procedure S2: IR, DSC, TGA experiments; Procedure S3: Evaluation of cytotoxicity; Figure S1. IR spectra of tea bag samples; Figure S2: DSC and TG curves for several tea bag samples; Table S1: Average size of tea bag particles after 5 min soaking in water; Figure S3: The scheme of “short-term” and “long-term” tea bags soaking experiments; Table S2: Concentration of polymer particles at 50 °C in water 1 h and 7 days after “short-term” tea bag soaking; Figure S4: Average size of polypropylene particles vs. soaking time; Figure S5: Average size of cellulose particles vs. soaking time; Figure S6: TEM images of polyamide and cellulose particles 1 and 4 days after soaking; Figure S7: Size distribution of cellulose particles 1 h and 7 days after soaking; Figure S8: Average size of polyamide and cellulose particles after soaking at an extract obtained via traditional tea brewing; Figure S9: Time-dependent electrophoretic mobility of polyamide, polypropylene and cellulose particles. Figure S10: Electrophoretic titration of water suspensions of tea bag-produced particles with a water suspension of cationic PVP.
