# Supplementary material for: Evolution of Microplastics Released from Tea Bags into Water
Source: Polymers (Basel). 2025 Oct 7;17(19):2700. doi: 10.3390/polym17192700 (PMC12526631; doi:10.3390/polym17192700)
Supplement: Supplementary file 1 [file polymers-17-02700-s001.zip › polymers-3901685-supplementary.pdf]

Supplementary Materials for

Evolution of microplastics released from tea bags into water

Alexander A. Yaroslavov [1, 2], Anna A. Efimova [1, 2], Tatyana E. Grokhovskaya [1], Anastasiia G. Badikova [1, 2], Vasily V. Spiridonov [1], Denis V. Pozdyshev [3], Sergey V. Lyulin [1, 2]*, Jose M. Kenny [2, 4]*

[1] *Department of Chemistry, M.V. Lomonosov Moscow State University, Leninskie Gory 1-3, 119991 Moscow, Russian Federation*.

[2] *Yaroslav-the-Wise Novgorod State University, B. St. Petersburgskaya str. 41, 173003 Veliky Novgorod, Russian Federation*.

[3] *Belozersky Research Institute of Physico-Chemical Biology, M.V. Lomonosov Moscow State University, Leninskye Gory 1-40, 119992 Moscow, Russian Federation.*

[4] *European Center for Nanostructured Polymers (ECNP), Loc. Pentima Bassa, 21, 05100 Terni, Italy*

[*] Corresponding authors: Sergey V. Lyulin (e-mail: sergey.v.lyulin@gmail.com) and Jose M. Kenny (e-mail: josekenny@yahoo.com)

Procedure S1. PVP synthesis.

PVP was synthesized by quaternization of poly-(4-vinylpyridine) with excess ethyl bromide, degree of polymerization equal to 600. The product was actually a copolymer containing 95 mol% of ethyl-quaternized pyridinium rings and 5 mol% of residual 4-vinylpyridine units (as found by IR spectroscopy).

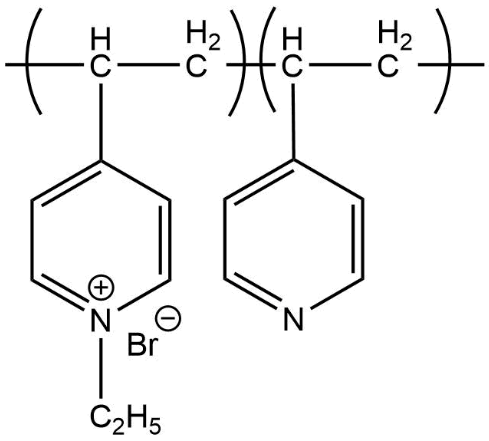

Procedure S2. IR, DSC, TGA experiments.

To conduct IR experiments, the studied samples were tableted in a KBr matrix; IR spectra were recorded in the absorption measurement mode. For the DSC experiments, aluminum crucibles were used that were hermetically closed with a lid by cold welding. The calorimetric curves were registered, heating the samples at a 10°/min rate; prior to the analysis, the baseline DSC was recorded. Thermogravimetric analysis was conducted in a TG50 cell at a temperature from 25 to 600 °C at a heating rate of 20 °/min in an air environment.

Procedure S3. Evaluation of cytotoxicity.

The standard analytical procedure is to incubate the test suspension (solution) with the cells, add the dye solution, dissolve the formazan crystals in dimethyl sulfoxide (DMSO), measure the optical density of the resulting solution and compare the result with the calibration curve in the absence of the test objects. Briefly, the day before the experiment, Caco-2 cells were seeded on a 96-well plate (Biofil, China) at a density 1.5×10^4 cells per well (in 0.1 mL DMEM/F12 (PanEco, Russia), 10% (v/v) fetal bovine serum (Hiclone, USA), 1% (v/v) L-glutamax (Sigma) and 1% (v/v) antibiotic solution (penicillin, streptomycin) (PanEco, Russia)). The next day, the culture medium was removed and 0.1 mL of the solutions of tea bag-produced polymer particles in phosphate-buffered saline (1xPBS) were added to the wells for 1 h. In the control wells (100% of surviving cells), the culture medium was replaced with 1xPBS. The solutions were then removed and the cells were cultured in 0.1 mL of complete medium for 24 hours. Subsequently, 0.1 mL MTT solution (0.375 mg/mL) was added to the culture medium for 4 hours. The medium was then removed, violet crystals of formazan were dissolved in 0.1 mL of DMSO and the optical density was measured at 570 nm on a VersaMax microplate reader (USA). The reference wavelength of 630 nm was used. The proportion of surviving cells was calculated as the ratio of the optical density in the well at a given concentration to that in the control well. All runs were performed in quadruplicate.

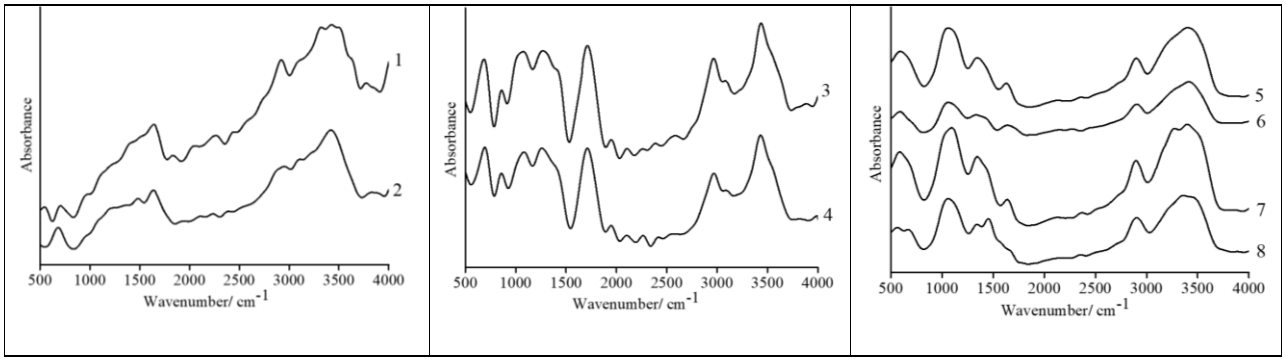

Figure S1. IR spectra of tea bag samples differed in their chemical composition: Sample I (1), Sample II (2), Sample III (3), Sample IV (4), Sample V (5), Sample VI (6), Sample VII (7) and Sample VIII (8).

The IR spectra of Sample I and Sample II contain a peak at 3300 cm^{-1} corresponding to the stretching vibrations of N-H groups in polyamides, and the peak at 2932 cm^{-1} associated with the ethylene sequence in polyamides (asymmetric stretching of CH_2 groups). Additionally, there are peaks in the range from 2000 to 500 cm^{-1}, which are characteristic for polyamide: at 1634 cm^{-1} (the Amide I band with the main contribution of the stretching vibrations of C=O group), 1535 cm^{-1} (the shoulder of the deformation vibrations of Amide II), 1371 cm^{-1} (the shoulder of the vibrations of Amide III and CH_2 groups) and 681 cm^{-1}, corresponding to the deformation vibrations of the N-H group. Thus, the IR spectra of Sample I and Sample II allowed to identify these polymers as polyamide.

The IR spectra of Sample III and Sample IV contain a peak at 808 cm^{-1}, corresponding to skeletal vibrations of C–C fragments in polyolefins, and peaks at 1166, 1376, 1456, 2920 cm^{-1}, corresponding to a set of stretching and deformation vibrations of C–H, CH_3, CH_2 groups in polypropylene. The IR spectra of Sample III and Sample IV were identified as polypropylene.

The IR spectra of Samples V–VIII contain a broad peak at 3331 cm^{-1}, characteristic of the stretching vibrations of the hydroxyl group in polysaccharides. This peak also includes inter- and intramolecular vibrations of hydrogen bonds in cellulose. The peak at 2894 cm^{-1} corresponds to vibrations of the hydrocarbon components CH_2 in polysaccharides. The peaks at 1633 cm^{-1} correspond to vibrations of water molecules absorbed by cellulose and its derivatives. The absorption bands at 1428, 1367, 1334, 1027 and 896 cm^{-1} belong to a set of stretching and deformation vibrations of the $-CH_2$ and -CH, -OH and C-O bonds in cellulose. Thus, in the IR

spectra of Samples V-VIII, absorption peaks characteristic of cellulose are observed. Based on the above data, the IR spectra of Samples V-VIII were assigned to cellulose.

**DSC curves**	**TG curves**
a Sample I 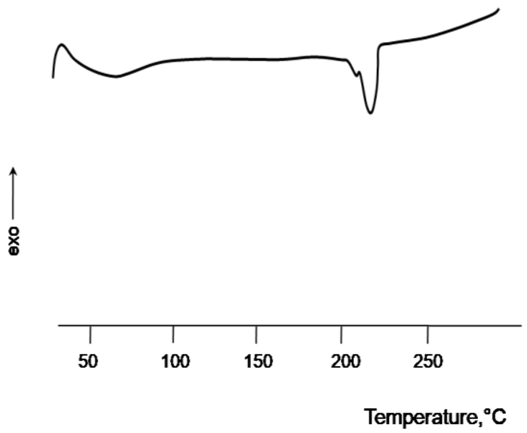	b Sample I 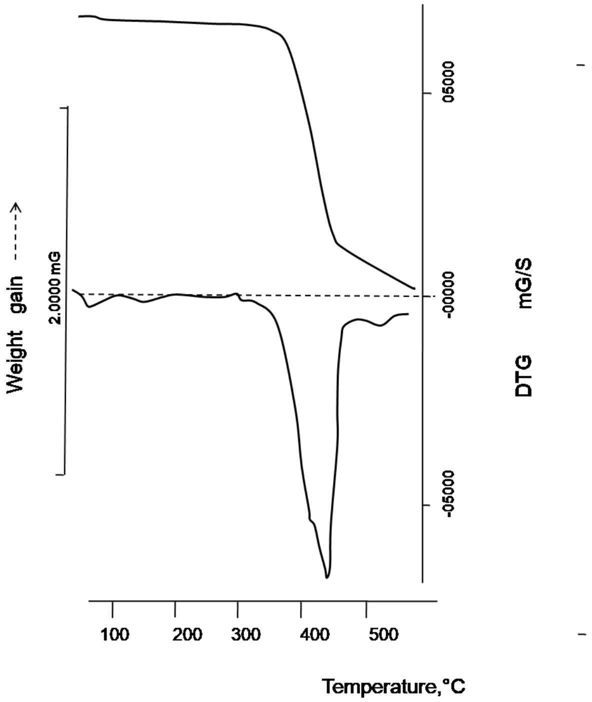
c Sample III	d Sample III

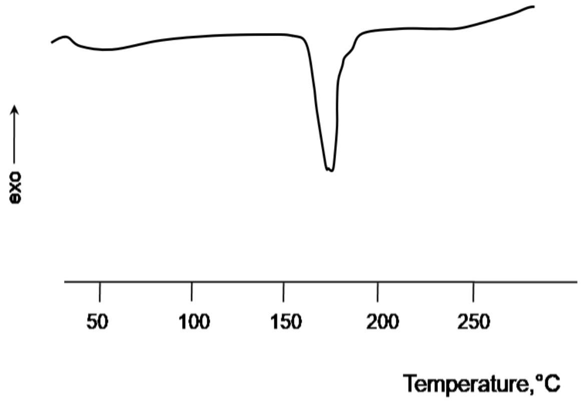

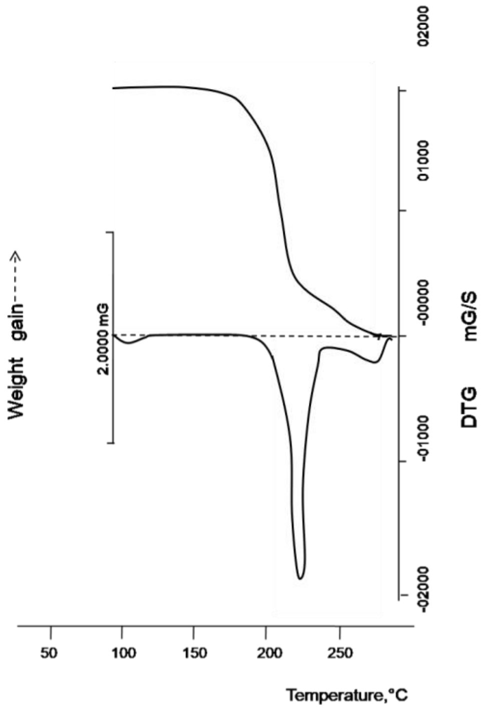

e Sample VI

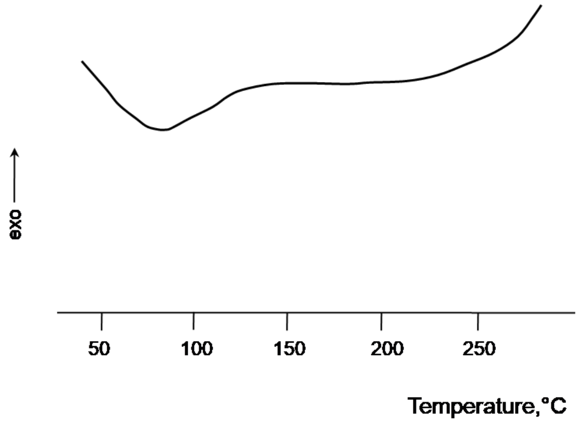

f Sample VI

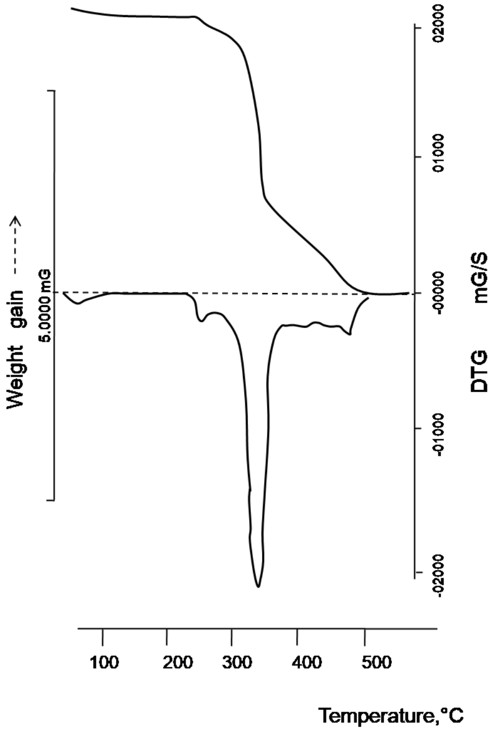

g Sample VII

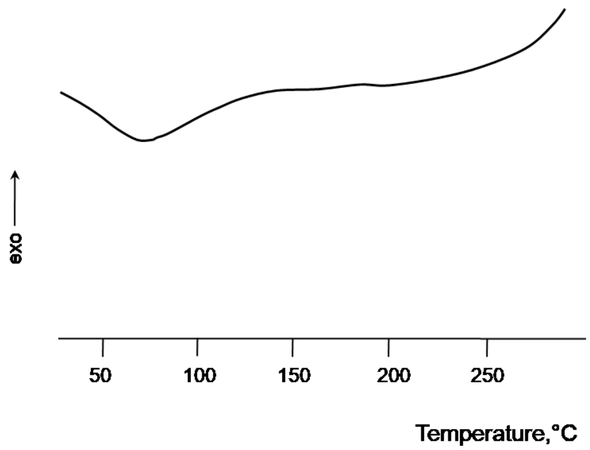

h Sample VII

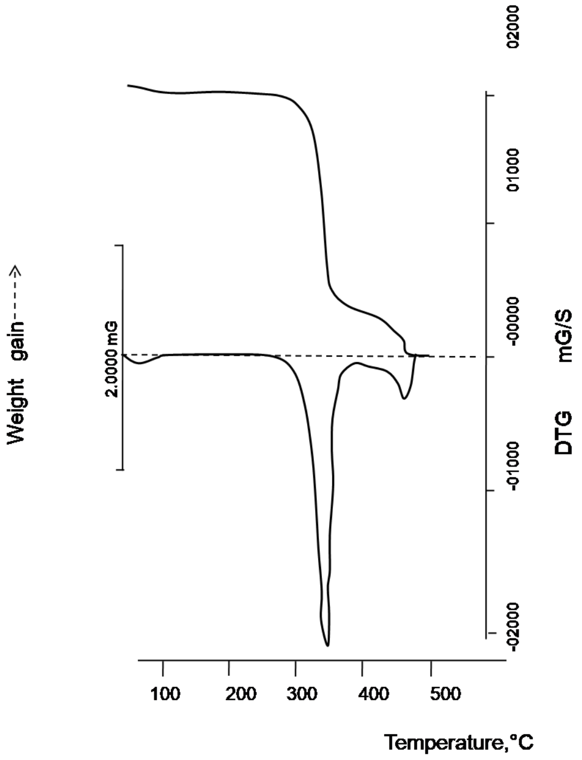

Figure S2. DSC (left columns) and TG (right columns) curves for several tea bag samples differed in their chemical composition: Sample I (a, b), Sample III (c, d), Sample VI (e, f), and Sample VII (g, h).

Table S1. Average size of tea bag particles after 5 min soaking in water.

Sample	Average size, nm		
	°C		
	20	50	100
I	Undetectable	Undetectable	600

III	Undetectable	Undetectable	Undetectable
VI	Undetectable	400	950

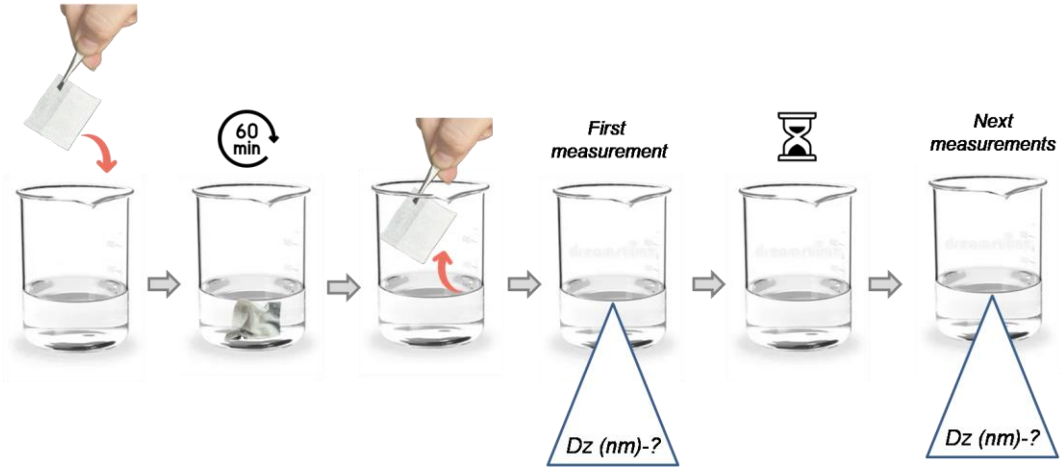

a

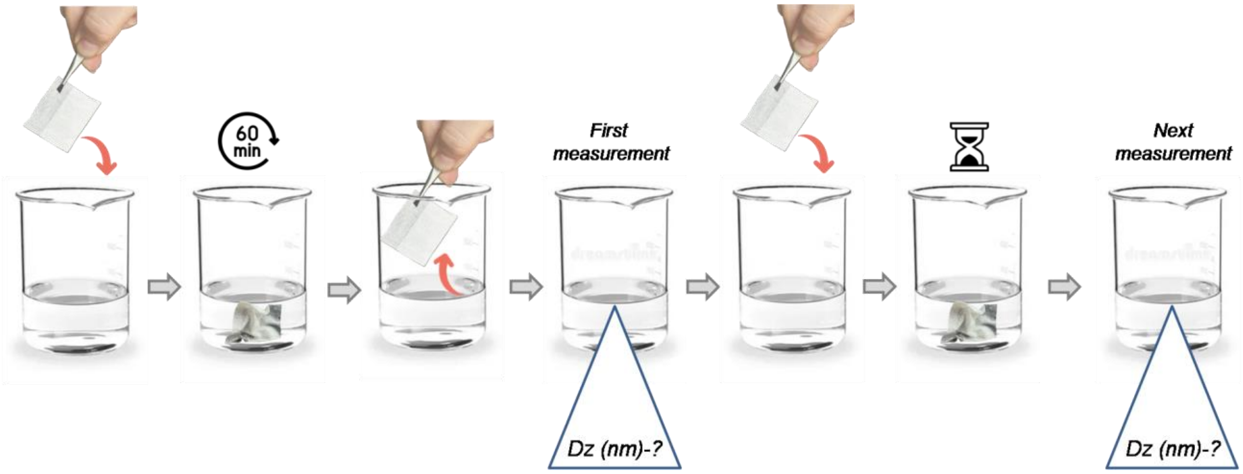

b

Figure S3. The scheme of “short-term” tea bags soaking (a) and “long-term” tea bags soaking (b) experiments.

Table S2. Concentration of polymer particles at 50 °C in water suspension 1 hour and 7 days after “short-term” tea bag soaking.

Sample	Concentration of polymer particle/10^9, L^{-1}	
	1 hour after soaking	7 days after soaking
I	14	23
VI	170	Undetectable

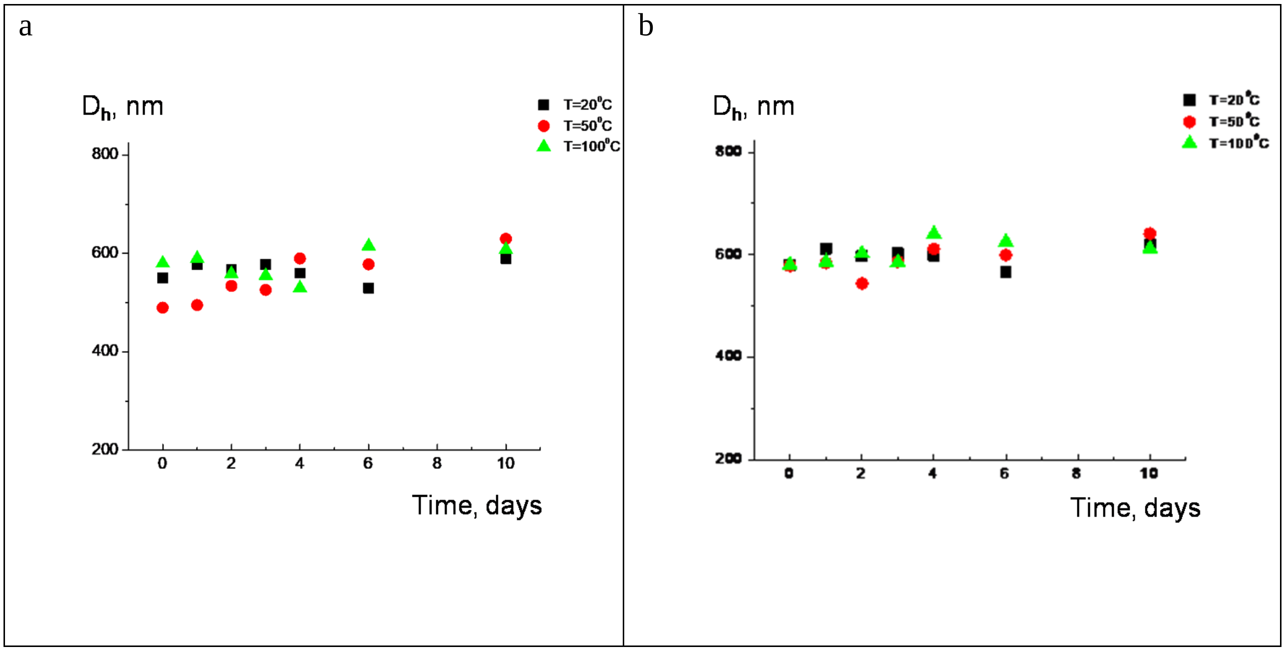

Figure S4. Average size of polypropylene particles (Sample III) vs. soaking time at 20, 50 and 100 °C during “short-term” tea bag soaking (a) and “long-term” tea bag soaking (b).

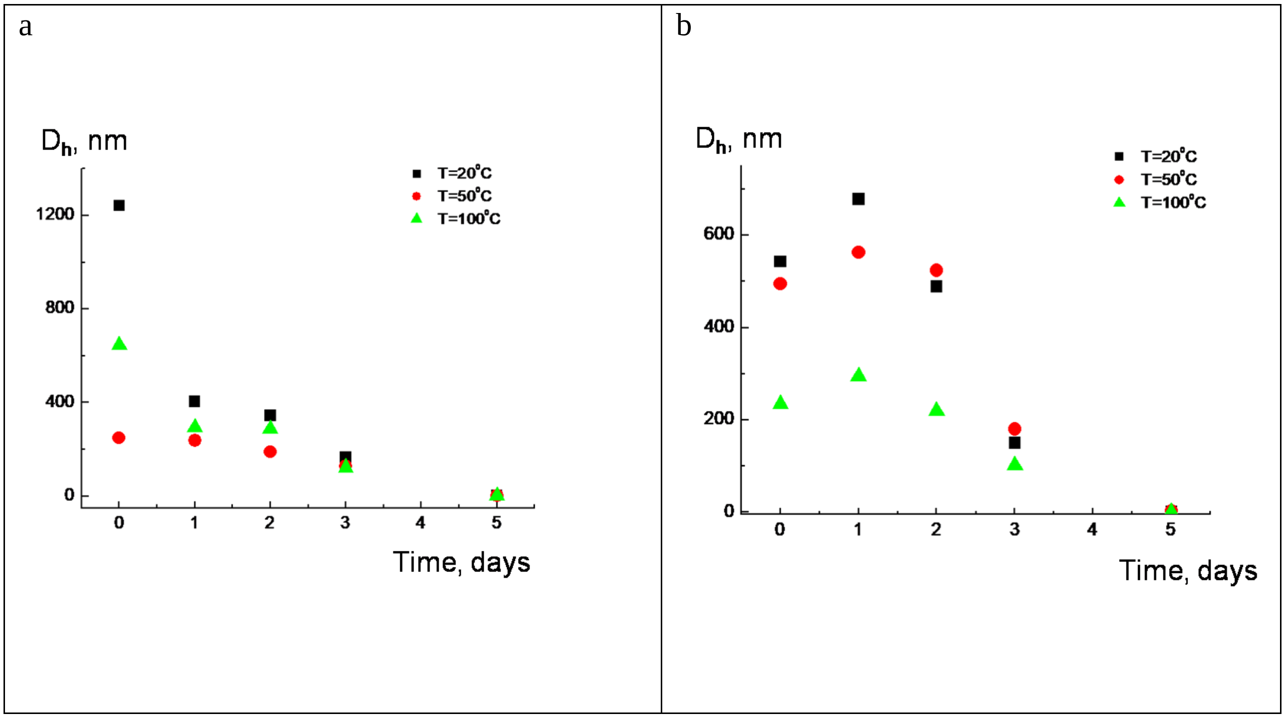

Figure S5. Average size of cellulose particles (Sample VI) vs. soaking time at 20, 50 and 100 °C during "short-term" tea bag soaking (a) and "long-term" tea bag soaking (b).

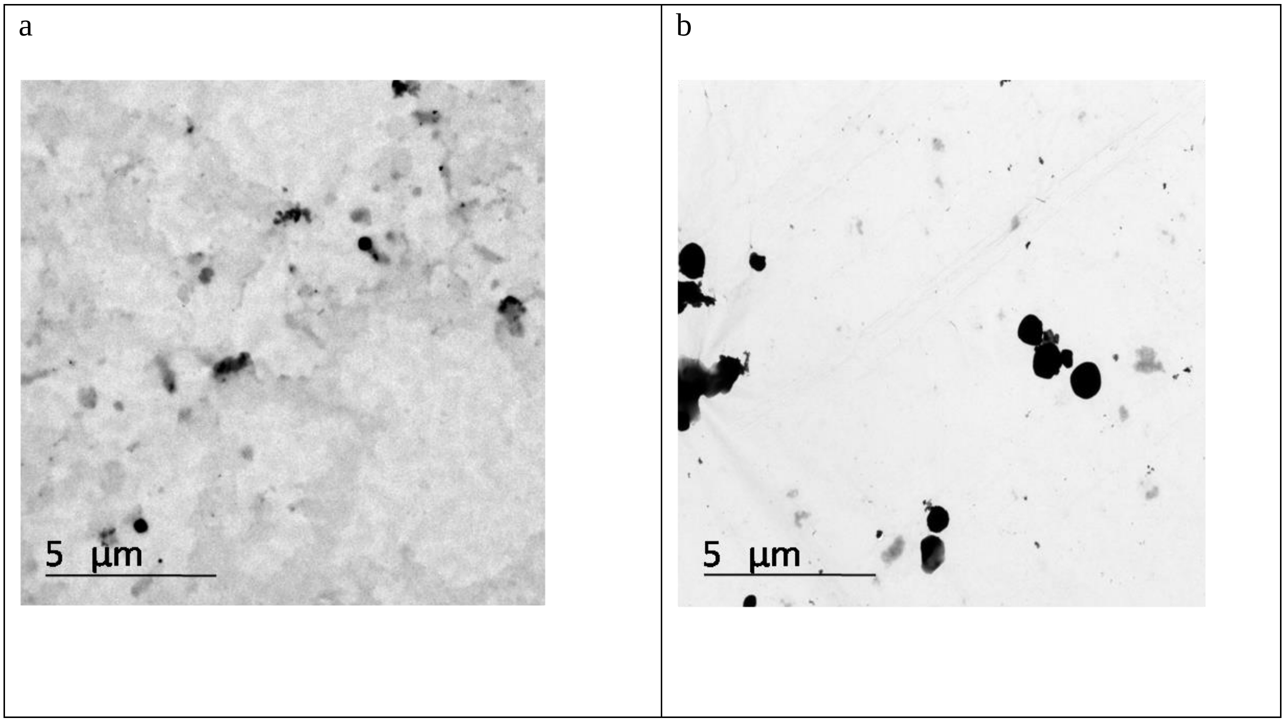

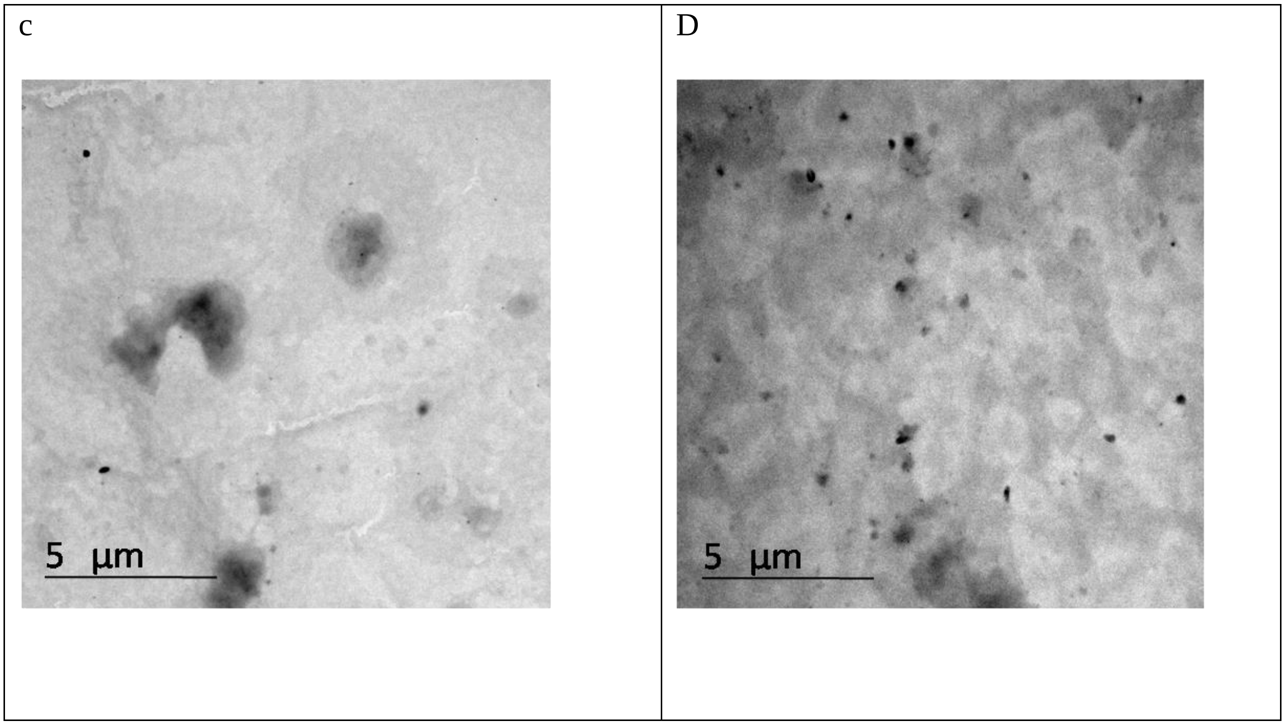

Figure S6. TEM images of polyamide (Sample II) (a, b) and cellulose (Sample V) (c, d) particles 1 (a, c) and 4 (b, d) days after "short-term” tea bags soaking; the particles were prepared in 100 °C aqueous solutions.

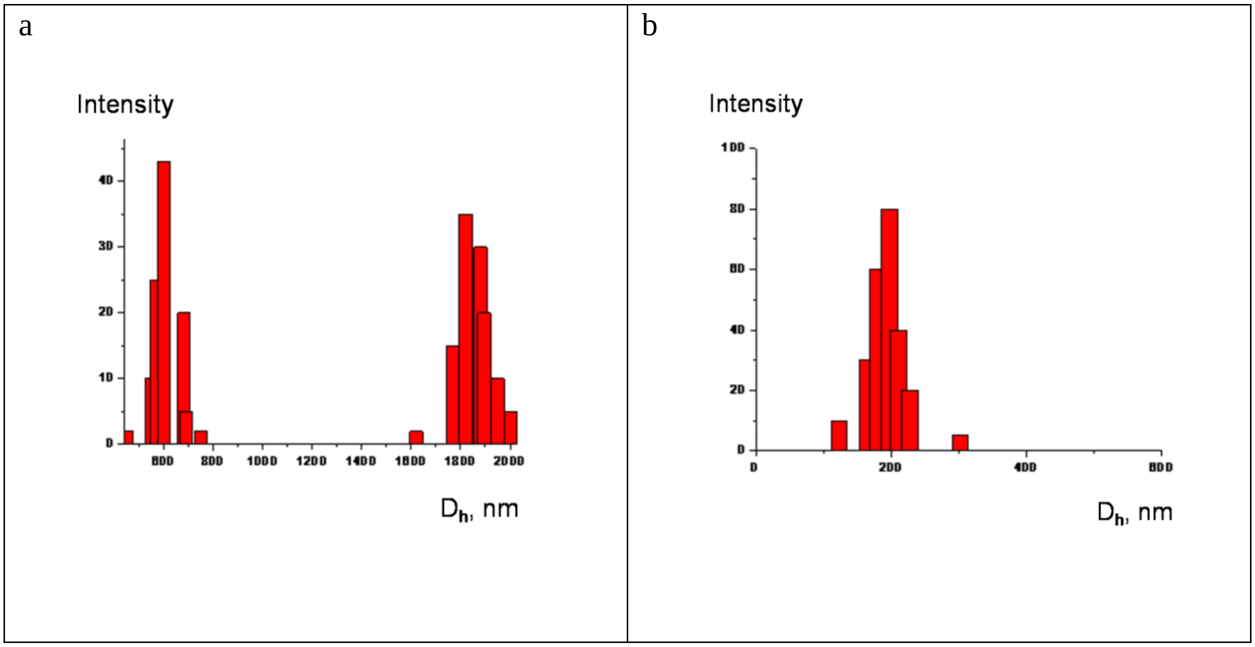

Figure S7. Size distribution of cellulose particles (Sample V) 1 hour (a) and 7 days (b) after soaking at 100 °C; the "short-term" soaking procedure.

a	b
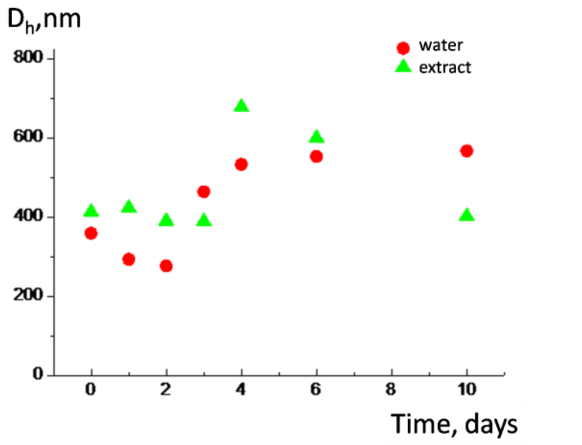	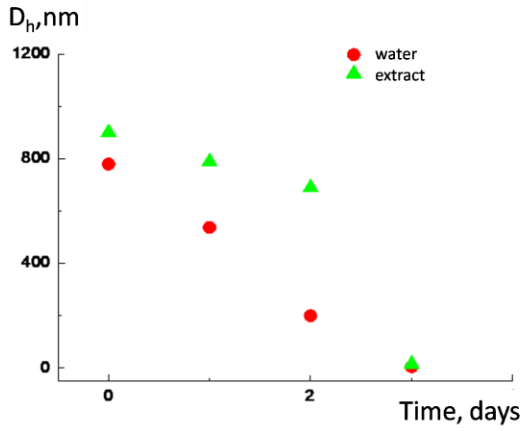

Figure S8. Average size of polyamide (Sample I) (a) and cellulose (Sample VII) (b) particles after "short-term" soaking at 50 °C at water and at an extract obtained via traditional tea brewing of the uncut tea bags.

a Sample II	b Sample III

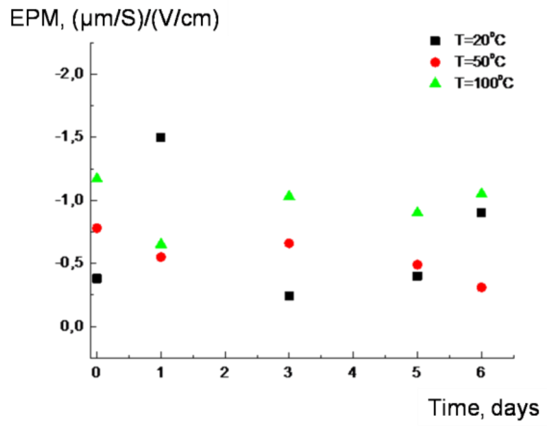

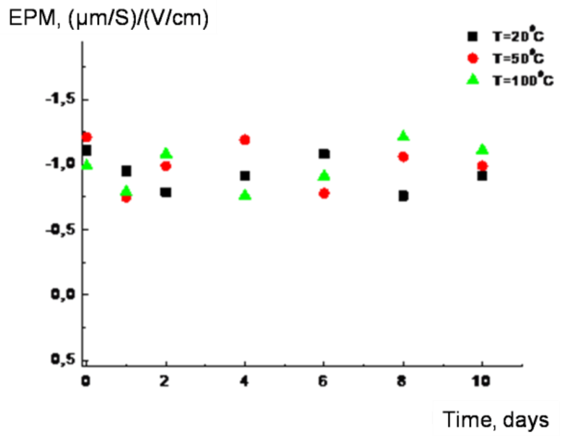

c Sample VIII

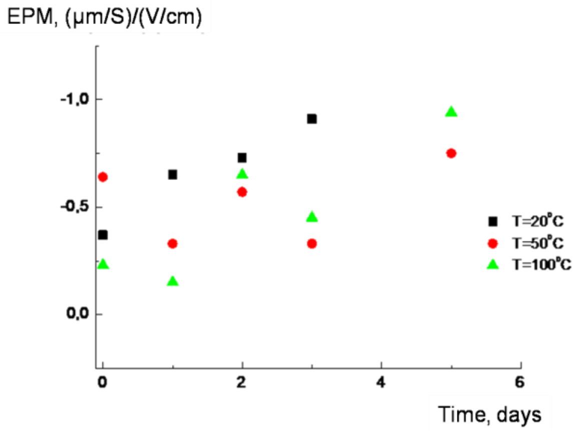

Figure S9. Time-dependent electrophoretic mobility of polyamide (Sample II (a)), polypropylene (Sample III (b)), and cellulose (Sample VIII (c)) particles at 20, 50 and 100 °C; for the "short-term" soaking procedure.

a	b
EPM, (µm/S)/(V/cm)	EPM, (µm/S)/(V/cm)

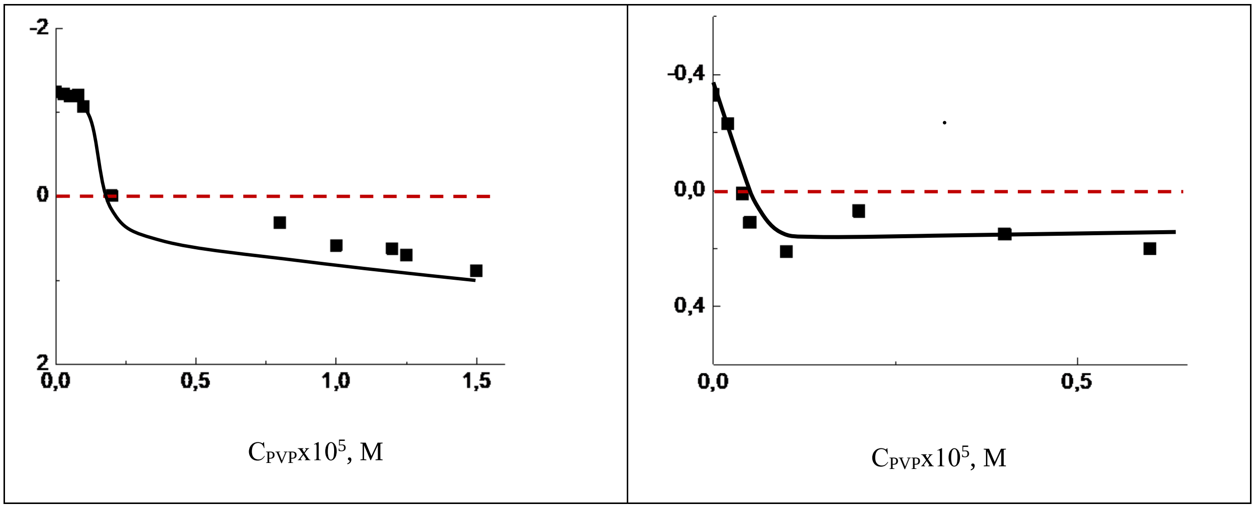

Figure S10. EPM of polyamide (Sample I) (a) and cellulose (Sample VI) (b) particles vs. molar concentration of PVP, 100 °C; the “short-term” soaking procedure.
